# The Molecular Mechanism of Metabolic Remodeling in Lung Cancer

**DOI:** 10.7150/jca.31406

**Published:** 2020-01-13

**Authors:** Ligong Chang, Surong Fang, Wei Gu

**Affiliations:** Department of Respiratory Medicine, Nanjing First Hospital, Nanjing Medical University. No. 68 Changle Road, Qinhuai District, Nanjing 210001,People's Republic of China.

**Keywords:** Lung cancer, Glycolysis, Lipid metabolism, Molecular mechanism

## Abstract

Metabolic remodeling is a key phenomenon in the occurrence and development of tumors. It not only offers materials and energy for the survival and proliferation of tumor cells, but also protects tumor cells so that they may survive, proliferate and transfer in the harsh microenvironment. This paper attempts to reveal the role of abnormal metabolism in the development of lung cancer by considering the processes of glycolysis and lipid metabolism, Identification of the molecules that are specifically used in the processes of glycolysis and lipid metabolism, and their underlying molecular mechanisms, is of great clinical and theoretical significance. We will focus on the recent progress in elucidating the molecular mechanism of metabolic remodeling in lung cancer.

## Introduction

Lung cancer (LC) is one of the most prevalent cancers and is the leading cause of cancer-related death worldwide. LC patients have low survival rates 1-2 with 56.2% of patients at stage III-IV at initial diagnosis; the five-year survival rate of LC is only 15% 3. The poor prognosis of LC is due to the difficulty in early diagnosis and the current poor understanding of the mechanisms underlying LC. Metabolic remodeling has been widely accepted as the basis for novel tumor biomarkers 4. Tumor cells, including LC cells, exhibit abnormal energy metabolism and significantly upregulated endogenous fatty acid metabolism. This upregulated metabolism, which is significantly different from the metabolism of normal cells, is called metabolic remodeling or reprograming. Metabolic remodeling takes place from the outset and throughout the development of LC, playing an important role in the evolution of LC 5.In this following sections we will explain metabolic remodeling and its importances from glycolysis metabolism and endogenous fatty acids metabolism in LC.

## Active glucose metabolism in lung cancer

### The significance of glycolysis

One century ago, Otto Warburg postulated that tumor cells still depend on glycolysis to produce adenosine triphosphate (ATP) when there is sufficient oxygen supply 6. Warburg speculated that this apparent aerobic glycolysis (termed the Warburg effect) might be related to impairment in the mitochondrial function of tumor cells 7. Generally, glycolysis is inferior to aerobic oxidation in terms of energy efficiency, the end product of aerobic glycolysisis lactic acid, which is fatal to cells. Therefore, some scholars regard aerobic glycolysis as a biological characteristic of tumor cells 8-9. However, glycolysis not only offers energy quickly in the case of mitochondrial damage and anoxic conditions 10, but can also force tumor cells to absorb abundant glucose to provide materials for lipid metabolism, nucleic acid metabolism and amino acid metabolism 11. The abundant intake of glucose by tumor cells, including LC cells, hinders nutrient supply to adjacent normal cells. Glycolysis can also induce deoxyribonucleic acid (DNA) mutation and the production of peroxide, both of which are beneficial for the proliferation and transfer of tumor cells 12.

There is still dispute over the mechanism underlying the Warburg effect. Warburg believed that the occurrence of a tumor is accompanied by mitochondrial damage; glycolysis plays an important role in energy metabolism 13. However, other studies have demonstrated that aerobic oxidation is normal and even activated in some tumor cells 14, and most ATPs needed by tumor cells come from aerobic oxidation 15. ATPs produced by glycolysis can account for 10-70% of ATP production in different tumors 16. Even more intriguing is that most tumor cells can maintain growth by adjusting metabolism according to the microenvironment. For example, in hematological malignancy, primary and superficial tumor cells of solid tumors primarily utilize aerobic oxidation, while deep cells of solid tumors utilize glycolysis to gain energy due to the anoxic environment. The reliance of these cell subsets on different metabolisms can form a metabolic symbiont through the metabolic cooperation of the shuttle mechanism of lactic acids 17-18. In a sense, the real significance of glycolysis is to provide intermediate products for other metabolisms 19.

Normal lungs contain the highest oxygen content, while nearly 50% of oxygen is used to make lactate; however, oxygen is rarely used to make proteins and fatty acids 20. Relative to other tissues, lungs consume more glucose and are the highest producers of glutamine. LC tissue exhibits increased glucose contribution to tricarboxylic acid cycle (TCA) cycle relative to normal lung tissue, while LC cells have different glycolysis rates and mitochondrial capacities. The metabolic phenotypes of LC cells can self-regulate based on the tissue environment 21. The proportion of hypoxic cells is consistently low in non small cell lung cancer (NSCLC), and there is no significant correlation between hypoxia and glucose metabolism in NSCLC 22-23. Recently, metabolic remodeling has also been found in fresh LC surgical tissues using Stable Isotope Resolved Metabolomics (SIRM) technology. These LC tissues showed increased levels of glucose-derived TCA cycle intermediates (e.g., lactic acid, alanine, succinic acid, glutamic acid) relative to normal lung tissue 24. Further, overexpression of pyruvate carboxylase (PC) and pyruvate was found in LC cells compared to normal lung tissues 25. In short, an increasing evidence that metabolic remodeling is profoundly activated during carcinogenesis and malignant progression in LC 26.

### Truncated TCA cycle and key molecules of glucose metabolism

Aside from ATP, TCA provides abundant intermediate products for the proliferation of tumor cells. Several studies have reported that TCA meets the needs of cell proliferation and invasion rather than providing ATP 27. Warburg speculated that impairment of mitochondrial function was related to abnormal expression of key enzymes on the respiratory chain, dysfunction of the electron transmission chain and abnormal expression of mitochondrial genes 28-29. In some tumors, damage of the respiratory chain induces a rise of reactive oxygen species (ROS) content. ROS can inhibit the key enzyme aconitase in TCA and thereby cause accumulation of citric acid in mitochondria. Citric acid can be decomposed into acetyl-CoA and acetoacetic acid. Acetyl-CoA is the raw material for synthesis of cholesterol and fat, which is called the truncated TCA cycle 30. The synthesized macromolecular substances are carried to the cytoplasm to participate in synthesis of lipids and proteins. Active glycolysis offers sufficient energies to NSCLC cells. Different NSCLCcells have different glycolysis levels.The key enzyme hexokinase 2 (HK2), phosphofructokinase (PFK), pyruvate kinase (PKM) and lactate dehydrogenase (LDH) have been reported to be overexpressed LC [31]. Inhibiting expression of glycolysis metabolic enzymes obviously suppressed LC cells proliferation via by the AKT signaling pathway 32.

The glycolysis level is related to apoptosis signal transduction. Disturbing glycolysis can significantly inhibit the malignant biological behavior of NSCLC cells 33. Metabolic remodeling of LC is related to drug resistance of epidermal growth factor receptor tyrosine kinase inhibitor (EGFR-TKI) 34. LC-driver genes (e.g. Kras and EGFR) can also lead to increased glucose metabolism in different modelling systems. Key drive gene mutant of LC cells exhibit increased glucose uptake 35. The mutant Kras; p53^fl^/^fl^ murine lung adenocarcinomas has similarities metabolic characteristics with human LC 36-37.KRAS mutations at codon-12 also had different metabolic remodeling and associated with different metabolomic profiles 38. recent researches display increased glucose uptake and aerobic glycolysis of KRAS-induced LC, Enhanced aerobic glycolysis lead to LC cells extracellular matrix microenvironment changes, and the microenvironment can facilitate the occurrence and development of KRAS-induced LC 39-41.Similar results were found in EGFR-driven LC, and tissue environment is an important determinant of tumor metabolic phenotypes 42.

### Active pentose phosphate pathway

**Figure 1:**
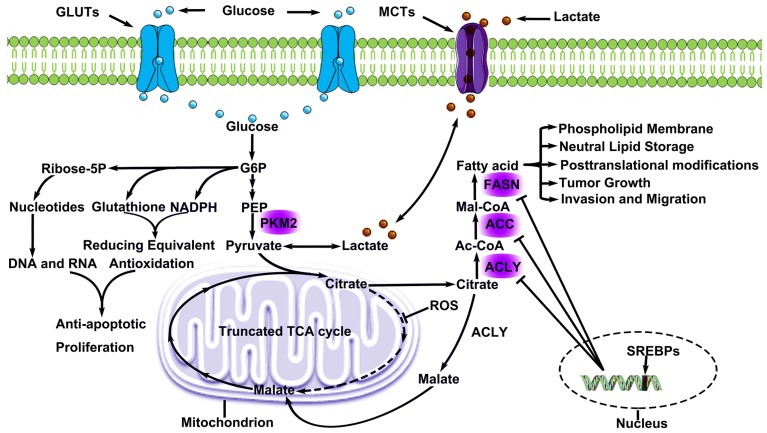
** Metabolic Remodeling in lung cancer**. Lung cancer cells consume large amounts of glucose via GLUTs and convert glucose to G6P, which involves in PPP metabolism and produced genetic substances, cytoskeleton and functional proteins. Pyruvate, a production by glucose, offers lactate for microenvironment and citrate for glycolsis. Lactate is excreted and absorbed through MCTs, and this phenomenon is known as lactic acid shuttle system. Citrate cannot participate in metabolism smoothly due to ROS inhibits a key enzyme (aconitase) activity in TCA. However, citrate participates in endogenous fatty acids metabolism. Some citrate is converted to malate and continues to participate in TCA. As a result, endogenous fatty acids metabolism offers lung cancer cells the energy for proliferation and invasion, and is regulated by three key regulatory enzyme, which are negatively regulated by SREBPs. G6P Glucose-6-Phosphate), MCTs (Monocarboxylate transporters), ROS (Reactive oxygen species),

To maintain their capacity for fast proliferation, tumor cells require not only ATP, but also genetic substances, a cytoskeleton, and functional proteins 43. During culture of tumor cells without glucose, alternative pathways of phosphopentose still synthesize the necessary substances for proliferation. Intermediate products produced by glycolysis activate the pentose phosphate pathway (PPP) through glucose-6-phosphate dehydrogenase (G6PDH) or transketolase TKT 44-45. The PPP that involves the catalyst, G6PDH, is the oxidative branch of PPP, while the PPP that involves the catalyst, TKT, is the non-oxidative branch of PPP. Products of glycolysis produce ribose 5-phosphate (R5P) through non-oxidative PPP, which is used in the synthesis of genetic substances. Meanwhile, PPP activation can induce abundant nicotinamide adenine dinucleotide phosphate (NADPH) and glutathione. NADPH can provide metabolic substrates and reducing equivalents for lipid metabolism and nucleic acid metablishm 46. An increasing number of studies have confirmed NADPH oxidase activity and expression related to malignant biological behavior of LC, and inhibition of NADPH oxidase function downregulates the proliferative and invasion of LC 47-48. NADPH oxidase can also supports glycolysis and promotes glutamine metabolism of LC 49.The glutathione metabolic system is directly participated in the metabolism of platinum drugs 50.NADPH and glutathione can both enhance anti-apoptosis in tumor cells and the PPP pathways of tumor cells in activated state. The activation of alternative pathways of phosphopentose plays an important role in the evolution of tumors **(Fig. 1)**.

## Accelerated endogenous fatty acid metabolism in LC

### The regulation of endogenous fatty acids metabolism

Fatty acids gained by normal cells from blood circulation are called exogenous fatty acids. Generally, exogenous fatty acids can meet the metabolic demands of normal cells, so normal cells (except liver, fatty tissue and breast tissue during lactation) rarely make de novo synthesis of fatty acids 51. Researchers discovered more than 50 years ago that the fatty acids required for the proliferation of tumor cells come mainly from de novo synthesis 52. Such fatty acids are also called endogenous fatty acids. Due to demands of tumor cells for malignant proliferation, exogenous fatty acid cannot meet cell proliferation requirements and so tumor cells activate the metabolism of endogenous fatty acids. Therefore, most key enzymes involved in the metabolism of endogenous fatty acids in tumor cells, such as ATP citrate lyase (ACLY), fatty acid synthase (FASN) and acetyl-CoA carboxylase (ACC), become highly activated 53. Fatty acids metabolic abnormalities could result in lung cancer. A meta-analysis included 31 studies found that inverse correlation between excess body weight (BMI>25 kg/m^2^) and lung cancer incidence 54-55. Another study has shown an association between total serum cholesterol (cut-off value; 5,3 mmol/L) and resectable NSCLC 56. Cholesterol via releasing EGFR from lipid rafts increases EGFR signaling activity 57. Endogenous fatty acids metabolisms were negatively correlation with EGFR expression and the fatty acids pathways may be valuable as a potential therapeutic target for lung adenocarcinoma 58.

Metabolic substrates (e.g. Ac-CoA) of endogenous fatty acids are acquired from the decomposition of citric acids by ACLY, while citric acid is produced by a truncated TCA cycle. Ac-CoA produces malonyl-CoA (Mal-CoA) under the catalysis of ACC while 9 Mal-CoAs aggregates into 16-C palmitic acids under the catalysis of FASN. Palmitic acids form essential lipids of cells under the catalysis effect of other specificity enzymes 59. ACLY is the bridge between glucose metabolism and lipid metabolism. In vivo and in vitro studies all prove the key role of ACLY in the evolution of tumor. High ACLY expression patients exhibited shorter life span than negative ACLY expression patients 60. Inhibiting ACLY disturbs NSCLC proliferation and ACLY can mediate occurrence of LC by participating in the metabolism of endogenous fatty acids. ACLY might be a new target for LC treatment 61-62. FASN is a key enzyme that catalyzes lipid synthesis and it has high expression in LC tissues 63-64.

High expression of FASN is closely related to proliferation and anti-apoptosis capacity, invasion and metastatic capacity of LC cells, as well as prognosis 65. ACC contains two subtypes (ACC1 and ACC2). ACC1 is the first key enzyme that catalyzes the denovo synthesis of fatty acids. Mal-CoA produced by ACC2 can repress the entrance of fatty acids into mitochondria for β oxidation of fatty acids, thus coordinating synthesis of liver fatty acids with β-oxidation and ketone synthesis. Inhibiting deficiency of ACC might cause complete blocking of the pathway for synthesis of fatty acids, which reflects that the pathways of fatty acid synthesis in LC cells are strictly regulated by ACC genes 66-68 **(Fig. 1)**.

### The rloe of endogenous fatty acids metabolism in LC

Synthesized fatty acids have extensive functions. β oxidation of fatty acids can produce Ac-CoA which offers raw materials for TCA. Moreover, fatty acids also participate in cell proliferation directly 69. The metabolism of endogenous fatty acids can also contribute to epithelial-mesenchymal-transition (EMT) regulation, thus influencing the invasive and metastatic capacity of LC cells 70. Lipid signals, such as prostaglandin E2 (PGE2), lysophosphatidic acid (LPA) and sphingosine-1-phosphate (S1P), can collect macrophages and immune cells, and stimulate the production of tumor capillaries 71-72. PGE2 can inhibit the activation of macrophages related to tumor, thus assisting tumor cells to escape from immunity monitoring 73. Phospholipid is an important component of the cytomembrane and organelle membrane. Reduction of phospholipids can influence the bioelectricity transduction of organelles with membranes and cells. Besides, phospholipids participate in the acetylation of proteins and other protein modifications after translation. Phosphatidylinositol, phosphatidylserine and lecithin can form the lipid raft structure to promote the activation of growth factor and participate in the activation of important signal pathways, such as phosphoinositide 3-kinase (P13K)/protein kinase B (AKT), Ras and Wnt 74. Multiple molecules can influence the activity of the metabolism of endogenous fatty acids. For example, sterol regulatory element binding proteins (SREBPs) are important transcription factors in sterol regulation and lipid synthesis and belong to one member of the basic helix-loop-helix (bHLH)-zip transcription factor family, with at least 3 spliceosomes (SREBP1a, SREBP1c and SREBP2). Under normal situations, SREBP and SREBP cleavage-activating protein (SCAP) form the composites in endoplasmic reticulum, and SREBP enters into the nucleus after cells are excited by stimulus signals. Meanwhile, the expressions of ACLY, ACC and FASN are regulated. Inhibiting pathways of lipid metabolism might be an alternative treatment for lung adenocarcinoma 75 (Fig. 1).

### The significance of endogenous fatty acids metabolism

Tumor cells choose de novo synthesis of fatty acids at the cost of abundant valuable ATPs and metabolites. Such a metabolic approach gives LC cells traits of fast proliferation and invasion. Two different kinds of drive-gene mutant (Kras^G12D^;P53^fl/fl^ and Kras^G12D^;LKB1^fl/fl^)mouse LC model have high rates of endogenous fatty acids metabolism 76, and the Kras^G12D^;LKB1^fl/fl^LC model has higher rates of endogenous fatty acids metabolism than Kras^G12D^;P53^fl/fl^^77^.This reveals that activated metabolism of endogenous fatty acids provides key substances for LC. Many anti-tumor drugs targeted at key enzymes of lipid metabolism have been developed based on the reported active lipid metabolism in LC cells. The discovery of high-efficiency anti-tumor targets based on lipid metabolism may become an edge in designing tools to defeat LC.

## Molecular mechanism related to metabolic remodeling of LC

### PI3K/AKT/mTOR signal pathway and LC

PI3K/AKT/mTOR is a typical signal pathway which is the focus of most current research. It mainly receives extracellular RTK (e.g. EGFR1/2/3/4, PDGFR, VEGFR, IGF-1R and HERs) signals to activate intra-cellular PI3K signals. PI3K signals activate the second receptor and the second receptor binds with the PH structure of AKT to activate AKT. AKT activates mTOR by inhibiting the formation of TSC1/TSC2 composite and PRAS40, an important negative regulatory factor in this pathway 78. mTOR participates in the transcription and metabolism of cell proteins by regulating many downstream factors, and thereby influencing cell growth and proliferation. It is reported in studies on LC that 50-73% of NSCLC patients have high expression of AKT and suffer poor prognosis 79-80, while 2-5% of NSCLC patients have mutations of PI3K and AKT. Moreover, 70% of NSCLC patients have an absence of the negative regulatory factor PETN of the PI3K/AKT/mTOR signal pathways, which further results in a poor prognosis 81-82. PI3K/AKT/mTOR pathway also participates in the regulation of EMT and glycolysis of LC 83-84.

**Figure 2:**
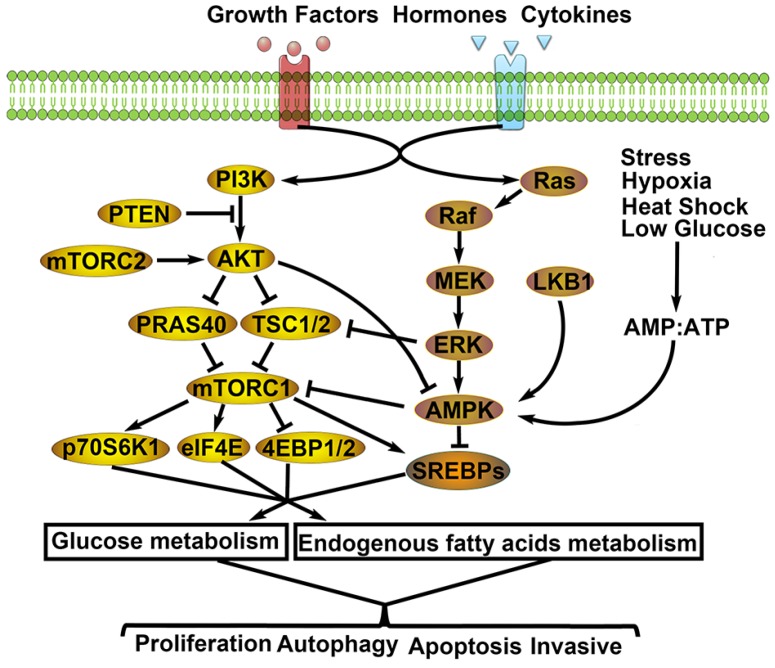
** Related molecular mechanisms of metabolic remodeling in lung cancer.** PI3K/AKT/mTOR and MEK/ERK/AMPK signaling pathways are both involved in metabolic remodeling of lung cancer cells. And, the two signaling pathways are regulated by extracellular signals (e.g. GFs, hormones, cytokines) to activate cascade response. Importantly, the interaction of the two signaling pathways affects glycolysis, TCA cycle, PPP, endogenous fatty acids metabolism. Consequently, the aggressive biological behaviors of lung cancer cells are activated by metabolic remodeling.

Inhibiting the PI3K/AKT/mTOR pathway can inhibit NSCLC proliferation of TKI, indicating that interrupting the PI3K/AKT/mTOR pathway might be a treatment strategy for TKI drug resistance 85-86. In summary, the PI3K/AKT/mTOR signal pathway participates in energy material metabolism, proliferation, autophagy, apoptosis and regulations of other biological functions of cells related to LC 87-90 **(Fig. 2)**.

### MEK/ERK/AMPK signal pathway and LC

Extracellular PTKs also can induce Raf activation when activating PI3K. The activated Raf then activates MEK, EPK and AMPK successively. Finally, the MEK/ERK/AMPK signal pathway is activated and participates in the occurrence and development of tumor cells. The MEK/ERK/AMPK signal pathway mainly perceives intracellular energy changes. Cells can activate AMPK automatically upon anoxia, ischemia, hunger and exercise. The activated AMPK can increase the supply of ATP and regulate the metabolic levels of glucose and lipids. Glycolysis inhibition sensitizes NSCLC with T790M Mutation to irreversible EGFR inhibitors via AMPK/mTOR/Mcl-1 pathway 91. Therefore, AMPK is also regarded as the monitor of energy level changes 92-95. According to studies on 3%-5% of NSCLC patients have mutation of BRAF 96 and some NSCLC patients have mutation of MEK, which could be used as the driving gene of NSCLC 97.

ERK can also participate in the formation of an inflammation microenvironment of LC cells 98. The MEK/ERK/AMPK signal pathway can participate in the regulation of drug resistance to NSCLC 99, and its retardants have been applied to stage-II clinical studies 100. MEK/ERK/AMPK pathway is important to the proliferation and apoptosis of LC cells 101-102 **(Fig. 2)**.

### Others molecular mechanisms for Metabolic Remodeling in LC

**Figure 3:**
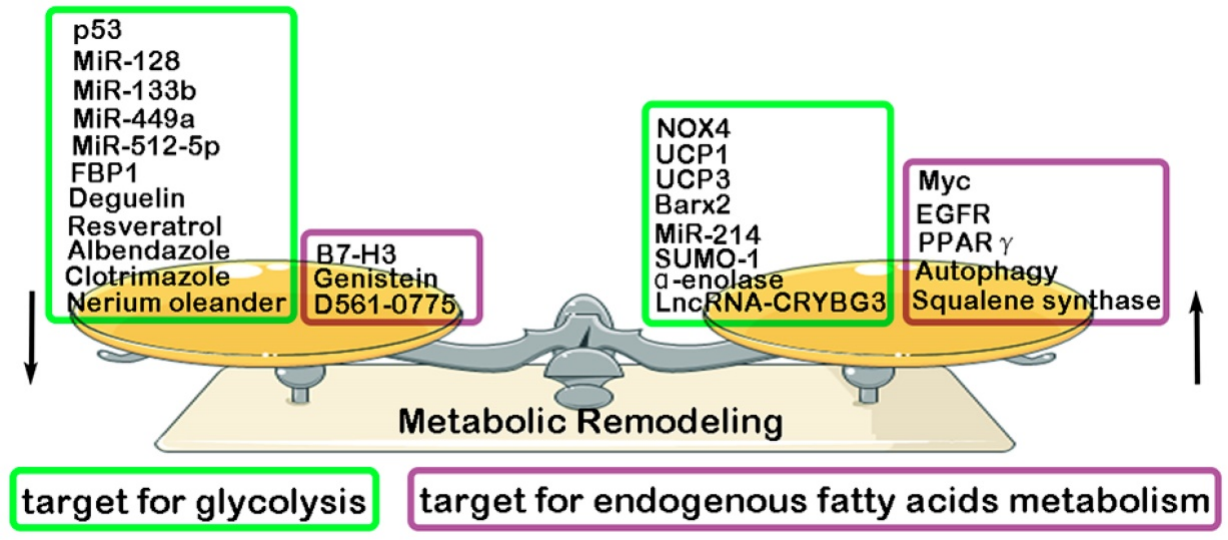
** Regulatory factors of metabolic remodeling in lung cancer.** Metabolic remodeling in lung cancer include glycolysis metabolism and endogenous fatty acids metabolism. Many negative/ positive regulatory factors involved in glycolysis and endogenous fatty acids metabolism.

Previous studies have found that some key molecules exerts its inhibitory effect in LC progression through down-regulating glycolysis, such as MiR-128 (inhibiting AKT) 108, MiR-512-5p (inhibiting p21) 109, MiR-133b (targeting PKM2) 110, MiR-449a (targeting LDHA) 111, p53 (targeting RRAD) 112, Albendazole (inhibiting HIF-1α) 113, Resveratrol(inhibiting HK2) 114, Deguelin (inhibiting HK2) 115, FBP1 116, Clotrimazole (targeting FDP) 117, Nerium oleander (targeting lactate) 118. However, some other molecules have been reported to play a promotive effect in glycolysis and glutamine metabolism of LC, such as MiR-214 (targeting HK2) 119, NADPH oxidase 4 (NOX4) (targeting PI3K/AKT pathway) 120, α-enolase (targeting PI3K/AKT pathway) 121, LncRNA-CRYBG3 (targeting LDHA) 122, BarH-like homeobox 2 (Barx2) (targeting Wnt/β-catenin pathway) 123, Small ubiquitin-like modifier 1 (SUMO-1) (targeting PKM2) 124, Uncoupling protein1/3 (UCP1/3) (targeting HK2 and PFK) 125. Compared with normal lung tissue, endogenous fatty acids metabolism is significantly enhanced in LC tissue. And, a high level of endogenous fatty acids metabolism has been reported to have a closely association with poor prognosis of LC patients 126-127. Recent studies have proven some key molecules that exert its inhibitory effect in endogenous fatty acids metabolism of LC, resulting in the suppression of LC malignant biological behavior. They are Genistein (inhibiting SCD1) 128, B7-H3 (targeting SREBP1) 129, D561-0775(inhibiting AMPK) 130. However, some other molecules were proven to exerts its promotive effect in endogenous fatty acids metabolism of LC, such as EGFR (targeting SCD1) 131, Autophagy 132, Myc (targeting COX and LOX pathway) 133, Squalene synthase (targeting TNFα) 134, PPARγ 135 **(Fig. 3).**

## Conclusions

Researchers have recognized many tumor characteristics through fighting tumors and formulating targeted treatments according to these characteristics. Tumor evolution is the consequence of both internal and external factors. The complexity of this evolution process is comparable with that of human evolution. For example, the first tumor of lung cancer may show significant damages. Although early screening, chemoradiotherapy, targeted treatment and immunotherapy have increased the diagnostic efficiency of lung cancer significantly, most patients with lung cancer develop unexpected progression of disease after multiple treatments. However, it is exciting that we have now recognized the metabolic difference between tumor cells and normal cells. These research conclusions reveal the relationship between abnormal metabolism and tumor evolution. The current study reviewed the molecular mechanisms of glycolysis metabolism and endogenous fatty acids metabolism of lung cancer, and offer a new opportunity for targeted tumor treatments.
